# Altered Expression of Mitochondrial Succinate Dehydrogenase Subunit D Influences Breast Cancer Progression

**DOI:** 10.3390/ijms27041722

**Published:** 2026-02-11

**Authors:** Jannatul Aklima, Israt Jahan, Khadiza Jahan, Utpal Barua, Shanjida Akter Touse, Shakera Ahmed, Ramendu Parial, Sunanda Baidya, Abu Shadat Mohammod Noman

**Affiliations:** 1 Department of Biochemistry & Molecular Biology, University of Chittagong, Chattogram 4331, Bangladesh; 2 EuGEF Research Foundation, Chattogram 4331, Bangladesh; 3 Department of Surgery, Chittagong Medical College, Chattogram 4203, Bangladesh; 4 Department of Pharmacology and Therapeutics, McGill University, Montreal, QC H3A 0G4, Canada

**Keywords:** breast cancer, SDHA, SDHB, SDHC, SDHD, METTL3

## Abstract

Mitochondrial succinate dehydrogenase subunits are differentially expressed in multiple tumor types, suggesting their role as a cancer type-dependent metabolic target. However, information on the expression of SDH subunits in breast cancer (BC), particularly in South Asian populations, remains scarce. So, we analyze the expression profile of all four SDH subunits in breast cancer patients from Bangladesh. qRT-PCR was carried out to analyze the mRNA expression of four SDH subunits in Luminal A, Luminal B, Her2+, and triple-negative breast cancer subtypes and the results were compared with The Cancer Genome Atlas (TCGA) database. All four succinate dehydrogenase subunits were significantly upregulated in tumor tissues when compared to controls and showed an alignment with the TCGA except SDHD, which was significantly downregulated in TCGA. Subtype-specific analysis demonstrated differential expression patterns. SDHD upregulation has also been connected to worse outcomes for patients which indicates its role in cancer progression. Furthermore, we found a significant upregulation of METTL3 in our patient cohort. Taken together, the elevated SDHD and METTL3 expression suggests a potential epigenetic mechanism-driven SDHD activation, highlighting its previously unreported role in breast cancer biology and revealing a distinct pattern of SDH dysregulation in the Bangladeshi breast cancer population.

## 1. Introduction

Mitochondria are essential organelles that maintain cellular balance through a complex network of metabolic and signaling pathways. In addition to making adenosine triphosphate, mitochondria also control biosynthetic pathways, redox homeostasis, immune signaling, and programmed cell death and survival [1,2,3,4,5]. One of the many enzymes that facilitate these central mitochondrial functions is succinate dehydrogenase (SDH), also known as mitochondrial Complex II. Mitochondrial SDH consists of four nuclear-encoded counterparts, including SDHA, SDHB, SDHC, and SDHD, whose integration into the mitochondrial inner membrane depends on various auxiliary factors, for instance, SDHAF1 and SDHAF2 [6,7].

The SDH complex is an essential part of the cellular metabolic integrity and redox homeostasis systems, and its structural or functional disruption is involved in diverse pathologies. Alteration in SDH subunits disrupt oxidative phosphorylation and the function of the TCA cycle, leading to succinate accumulation, a metabolic disturbance related to pseudohypoxia, epigenetic reprogramming, and oncogenesis [8,9,10,11,12,13,14,15]. As such, the SDH has long been regarded as a tumor suppressor, and its dysfunction was found to be linked to a variety of cancer types, including renal cancer, hereditary pheochromocytoma/paraganglioma and pituitary adenoma [12,15,16,17,18]. However, recent research suggests that the position of SDH in malignancy is more nuanced than the loss-of-function paradigm. Indeed, depending on the tumor nature and context, SDH subunits can have distinct expression profiles or have a varied pro-tumorigenic behavior [19,20].

Notably, elevated SDHA expression has been identified in several malignancies, including ovarian, breast and uveal melanoma where it stimulates mitochondrial bioenergetics and enhances tumor proliferation, migration, and metabolic flexibility [21,22,23,24]. More promisingly, in colorectal and lung adenocarcinomas, higher SDHA action is linked to better response to immunotherapy [19,25]. Again, in multiple myeloma, elevated SDHA plays a role as a good prognostic factor [26]. In these contexts, SDHA’s tangled actions underscore its tissue and context-specific patterns. In contrast, low or mutated SDHA results in augmented succinate formation, impairing mitochondrial respiration and contributing to oncogenic metabolism [8,19,20,27,28,29,30]. The same divergency can be seen with SDHB, which has been described as an extensively studied tumor suppressor that is inactivated in breast cancer, hepatocellular carcinoma, colorectal cancer, paraganglioma and renal carcinoma, in addition to many other tumors [31,32,33,34,35]. Nevertheless, the selective induction of SDHB in hepatocellular carcinoma decreases bioenergetic signaling and blocks cell migration and cell growth [36,37].

SDHC and SDHD mutations have also been linked to cancer. SDHC is often suppressed in aggressive, basal breast tumors and colorectal cancer [31,38], and reduced expression is associated with the emergence of the epithelial to mesenchymal transition [38,39]. Similarly, as SDHC, the lack of SDHD in hereditary pheochromocytomas and paragangliomas has been linked to tumor initiation through succinate gathering and aberrant signaling pathways [40,41]. Furthermore, downregulation of SDHD is linked to the tumorigenesis through various mechanisms [11,42,43]. However, the role of SDHD overexpression in conferring adaptive or oncogenic benefits remains unresolved, as the evidence on its upregulation in solid tumors is sparse and inconclusive. Nevertheless, upregulation due to epigenetic variations may contribute to altered cellular metabolism and cancer [44]. These variations across cancer types suggest that SDH subunits may be involved in subtype-specific or population-specific metabolic reprogramming rather than functioning uniformly as suppressors or promoters of tumorigenesis.

Breast cancer (BC) is a heterogeneous disease that comprises several molecular subtypes, including Luminal A, Luminal B, Human epidermal growth factor Receptor 2 positive (Her2+), and Triple-Negative Breast Cancer (TNBC), which show intrinsic differences in genetics, metabolism, and treatment strategies [45,46,47,48]. Substantial evidence suggests that mitochondrial dysfunction and selective alterations in oxidative metabolism drive subtype-specific progression and resistance in breast tumors [49,50]. However, the expression patterns of SDH subunits in breast cancer are not well-characterized, particularly in ethnically diverse and underrepresented populations such as those in South Asia. Most previous large-scale genomics studies on breast cancer, such as The Cancer Genome Atlas, were collected from Western cohorts and may not fully capture disease heterogeneity [51,52]. Consequently, the extensive metabolic landscape of breast tumors in countries such as Bangladesh remains predominantly unexplored. We address this gap by analyzing the expression profiles of the four SDH subunits—SDHA, SDHB, SDHC, and SDHD—in the principal molecular subtypes of breast cancer within a cohort of Bangladeshi individuals and then cross-compare these findings with data from the TCGA. We also identified the epigenetic link of altered expression in our population. Again, by emphasizing the subtype-specific and population-specific alterations in SDH expression, this analysis can improve our understanding of the metabolic heterogeneity of breast cancer and will help to clarify the metabolic adaptations that facilitate breast cancer progression. More broadly, our study could identify new metabolic targets and prognostic indicators in the future for breast cancer that are influenced by mitochondrial functions.

## 2. Results

### 2.1. Clinico-Pathological Study of Breast Cancer Patients

This pilot study was conducted in Chattogram, the second-largest and most densely populated city in Bangladesh. This city is a prime target area for cancer screening research. All data herein were collected from the cohort of breast cancer patients, Chittagong Medical College Hospital (CMCH), Bangladesh, under protocol codes. Their tumor grade and intrinsic molecular subtypes were confirmed by histopathology and immunohistochemistry (IHC). A total of twenty five breast cancer samples were included in this cohort of patients and were under investigation for alterations of four succinate dehydrogenase subunit genes. The dataset analyzed in this study consisted of four molecular subtypes: Luminal A (*n* = 6), Luminal B (*n* = 6), Her2+ (*n* = 6), and TNBC (*n* = 7).

### 2.2. SDH Subunits Show Differential Expression in Breast Cancer Patients

**Figure 1:**
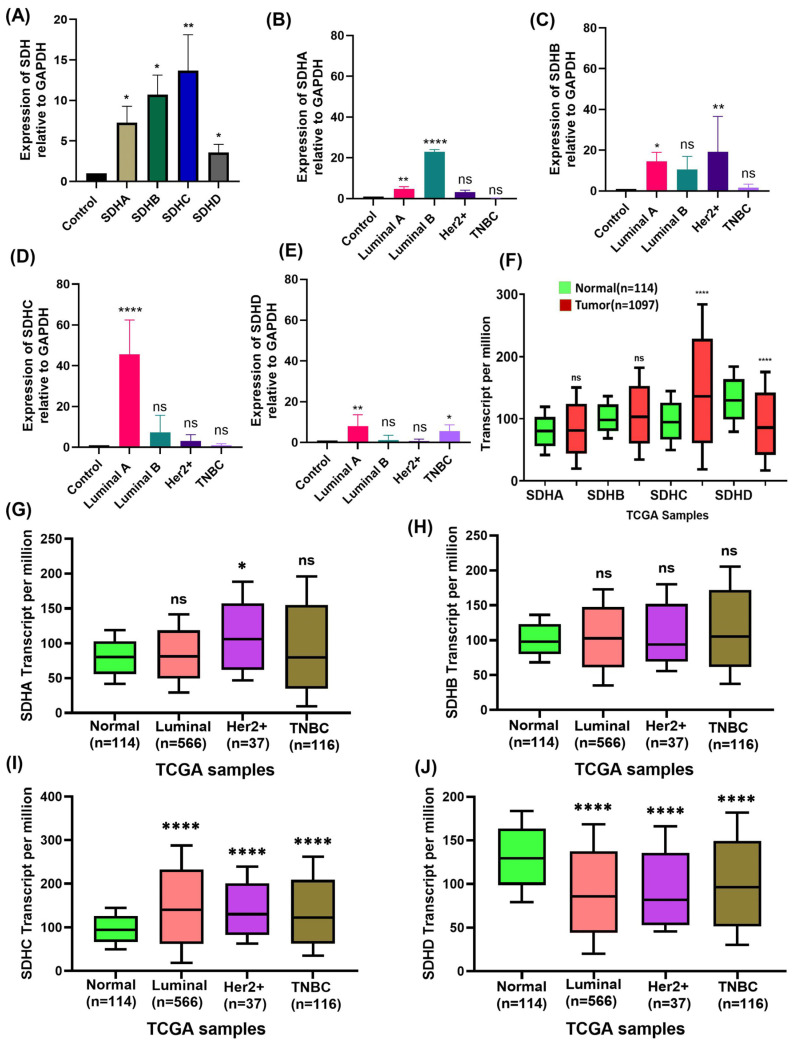
SDH expression in breast cancer tissues. (**A**) mRNA expression levels of SDHA, SDHB, SDHC, and SDHD relative to adjacent normal tissues (control) were measured by the 2^−ΔΔCt^ method after RNA expression was detected and quantified with GAPDH as a control. (**B**–**E**) The expression levels of (**B**) SDHA, (**C**) SDHB, (**D**) SDHC, and (**E**) SDHD in breast cancer subtypes (Luminal A, Luminal B, Her2+ and TNBC) from our patient cohort. (**F**) mRNA levels of the four SDH genes in breast cancer tissue vs normal tissue in TCGA. (**G**–**J**) Differential expression of (**G**) SDHA, (**H**) SDHB, (**I**) SDHC, and (**J**) SDHD in the subtypes Luminal A, Luminal B, Her2+, and TNBC in TCGA data. Data are expressed as mean ± SD and *p* values < 0.05 are considered significant; * *p* < 0.05; ** *p* < 0.01; **** *p* < 0.00001; ns, not significant.

To measure SDH genes expression, we performed quantitative real-time PCR of breast cancer tissues and adjacent non-cancerous tissues as normal. Our results demonstrated significantly higher expression of SDHA, SDHB, SDHC and SDHD mRNAs in breast cancer specimens compared to controls (Figure 1A). In terms of specific subtypes of breast cancer, SDHA mRNA was upregulated in Luminal and Her2+ tumors; however, this difference was not statistically significant in Her2+ types (Figure 1B). While upregulation was observed in both Luminal cases, Luminal B cells demonstrated a strong upregulation (*p* < 0.001) (Figure 1B). On the contrary, TNBC samples showed a non-significant downregulation compared with the control (Figure 1B). Next, we analyzed the expression of SDHB in four different cancer subtypes. While SDHB was significantly upregulated in Luminal A and Her2+ tumors (Figure 1C), it was not found to be significantly overexpressed by Luminal B and TNBC types (Figure 1C). Additionally, the SDHC expression in breast cancer subtype revealed differences, as all the four subtypes showed upregulation, but only Luminal A type was highly significant (Figure 1D). Evaluation of SDHD expression in breast cancer tissues showed increased expression in Luminal A, Luminal B and TNBC, but a non-significant decrease in Her2+ cases (Figure 1E). Among Luminal A, Luminal B, and TNBC cases, only Luminal A and TNBC showed significant increases, whereas Luminal B was non-significant (Figure 1E). Thus, across the four subtypes of breast cancer, our data reveal a differential expression pattern of SDHA, SDHB, SDHC, and SDHD mRNAs.

Further analysis of the TCGA database revealed that the expressions of SDHA, SDHB and SDHC were increased in breast cancer tissues compared to control; however, the difference was statistically significant only in SDHC (Figure 1F). On the other hand, SDHD was significantly downregulated in breast cancer with respect to normal control (Figure 1F). We then analyzed subtype-specific expression of the SDH genes in the TCGA data. In the case of SDHA, the Luminal and TNBC groups showed a non-significant higher expression with relatively similar median value as the normal group. However, the Her2+ group had significantly higher SDHA expression than normal, with the median value slightly above 150 transcripts per million (Figure 1G). We found similar SDHB expression levels in the Luminal, Her2+, and TNBC groups with the median value around 100 transcripts per million. All the values showed an upregulation trend except Her2+, as its median value is slightly lower than normal but there was no statistically significant difference in SDHB expression levels between the Luminal, Her2+, and TNBC subtypes (Figure 1H). SDHC expression was significantly higher in the Luminal, Her2+, and TNBC groups, with median values around 150–250 transcripts per million, compared to the normal group, which had a median around 100 transcripts per million (Figure 1I). Thus, we observed a marked elevation in SDHC expression across all breast cancer subtypes. In contrast, the normal group showed the highest SDHD expression, with a median of approximately 150 transcripts per million (Figure 1J). For the Luminal, Her2+, and TNBC subtypes, SDHD expression was found to be significantly downregulated compared to normal (Figure 1J). In both patient cohorts and TCGA data, SDHA and SDHC showed increased expression in the Luminal and Her2+ subtypes. On the other hand, SDHD was found to be downregulated in the Her2+ subtype in both patient cohort and TCGA data. All other case variations were observed between the patient cohort and TCGA data. Although both datasets indicate similar trends, the discrepancies may be due to differences in experimental approaches, sample size, subtype composition, and, more specifically, epigenetic variations within the patient cohort, as TCGA data were primarily focused on Western cohorts. Furthermore, the TCGA database has a wider range of expression levels in all the cases, which may be due to the large number of patients in the cohort.

**Figure 2:**
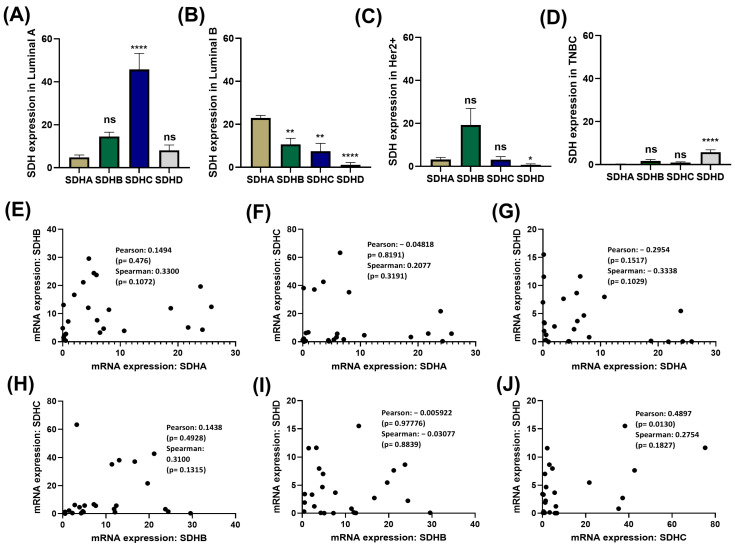
SDHx expression variation and correlation. (**A**–**D**) SDHA, SDHB, SDHC, and SDHD expression levels in various subtypes of breast cancer: (**A**) Luminal A, (**B**) Luminal B, (**C**) Her2+, and (**D**) TNBC. (**E**–**J**) SDH gene pairs, (**E**) SDHA vs. SDHB, (**F**) SDHA vs. SDHC, (**G**) SDHA vs. SDHD, (**H**) SDHB vs. SDHC, (**I**) SDHB vs. SDHD, and (**J**) SDHC vs. SDHD were analyzed for correlation and co-expression. Strong correlations that were statistically significant were not found. A *p* value of less than 0.05 is deemed statistically significant, and data are displayed as mean ± SD: * *p* < 0.05; ** *p* < 0.01; **** *p* < 0.00001; ns, not significant.

To further confirm this, we then analyzed the correlation and co-expression among the SDH genes (Figure 2). In our patient cohort, we observed significant variations among the four subunits’ expression in four different cancer subtypes (Figure 2A–D). Across all SDH gene pairs (SDHA-SDHB, SDHA-SDHC, SDHA-SDHD, SDHB-SDHC, SDHB-SDHD, and SDHC-SDHD), comparisons show weak, non-significant correlations in some cases only (Figure 2E–J). Overall, the SDH subunits expression levels do not show strong or reliable co-variation in these datasets. We further confirm these findings in TCGA data which showed weak non-significant correlation among the pairs of SDH subunits (Supplementary Figure S1A–F). Overall, SDH subunits show subtype-specific dysregulation in breast cancer with only weak, non-significant co-expression, suggesting they are not tightly co-regulated and may contribute independently to breast cancer biology.

### 2.3. SDH Subunits Expression and Overall Survival in Breast Cancer

**Figure 3:**
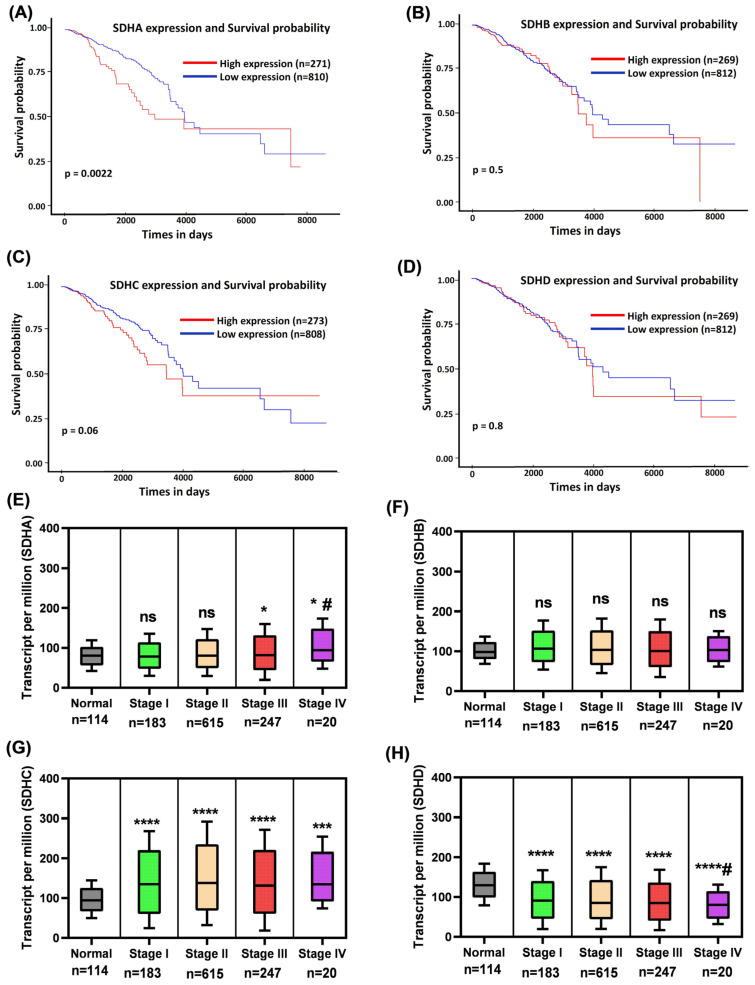
Survival analysis based on SDHx gene expression. Kaplan–Meier survival analysis was performed to assess survival probability in breast cancer patients based on the expression of (**A**) SDHA, (**B**) SDHB, (**C**) SDHC, and (**D**) SDHD. The relationship between gene expression and survival showed variability. (**E**–**H**) Differential expression of (**E**) SDHA, (**F**) SDHB, (**G**) SDHC, and (**H**) SDHD in different stages of breast cancer in TCGA data. Data are expressed as mean ± SD and A *p* value < 0.05 was considered statistically significant. * *p* < 0.05 vs. Normal and # *p* < 0.05 vs. Stage I; * *p* < 0.05; *** *p* < 0.0001; **** *p* < 0.00001; ns, not significant.

We have examined whether the differential expression of SDH subunits correlates with patients outcomes. Dysfunction of the succinate dehydrogenase complex in breast cancer impairs mitochondrial metabolism and the TCA-cycle/ETC coupling, leading to the accumulation of succinate and hypoxia-like signaling. This, in turn, encourages more aggressive tumor behavior and is increasingly associated with worse patient survival outcomes [8,10,24,39]. The expression of SDH subunits can differ considerably between cancer types and has been demonstrated to affect tumor biology and patient survival in breast cancer [24,31,39]. This association underlines the complex relationship between SDH expression and patient survival in cancer. The Cancer Genome Atlas (TCGA) cohort further supports the critical role of SDH subunits expression in patient survival. High expression levels of SDHA, SDHB, and SDHD were significantly associated with poor survival probability, underscoring the negative impact of these subunits on patient survival (Figure 3A,B,D). Conversely, low SDHC expression was also associated with decreased survival, emphasizing the importance of this subunit in patient outcomes (Figure 3C). Subtype-specific analyses revealed that these survival correlations hold for most breast cancer subtypes (Supplementary Figure S2A–D), with the worst outcomes in TNBC cases for the overexpression of all four SDH subunits. This suggests that while SDH subunits’ expression generally affects survival across most breast cancer types, TNBC may exhibit unique mechanisms that influence the adverse impact on survival. In conclusion, the expression of SDH subunits plays a crucial, differential role in the survival of breast cancer patients. Both high and low expression of specific subunits, such as SDHA and SDHC, respectively, are linked to poorer survival outcomes, highlighting the complex role of mitochondrial function in breast cancer progression and patient survival. Further studies are needed to better understand the mechanistic pathways by which these subunits influence tumor behavior and to explore their potential as therapeutic targets in breast cancer treatment.

To further assess their involvement in cancer progression, we analyzed SDHx subunit expression across breast cancer stages in TCGA samples. SDHA levels were elevated at all four stages, but the increase was statistically significant only in stages III and IV. When we compared stages, only stage I versus stage IV showed a significant difference (Figure 3E). Similarly, SDHB and SDHC were also higher than in normal tissue at all stages, but these changes were statistically significant for only SDHC (Figure 3F,G). SDHD showed the opposite pattern; its expression was significantly decreased in all stages of breast cancer, with an additional significant difference between stage I and stage IV (Figure 3H). Together, these findings further support a role for SDH subunits in breast cancer progression.

### 2.4. Differential Expression Driven by Copy Number Alterations (CNAs)

**Figure 4:**
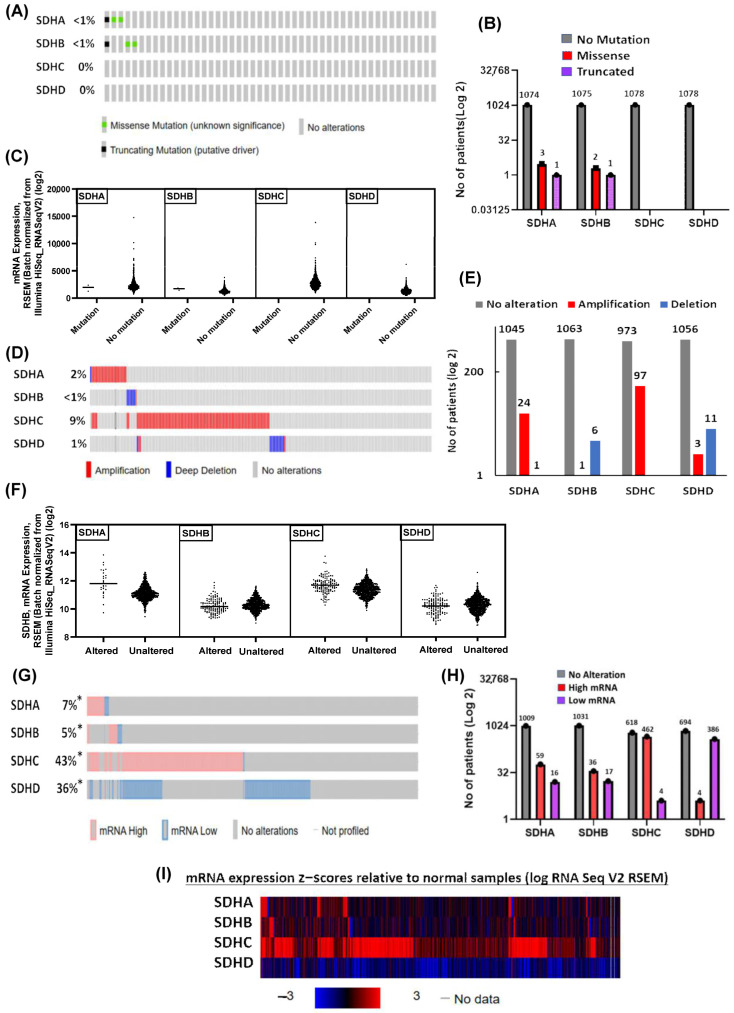
SDHx mutations and copy number alterations. (**A**) Oncoprint visualization of genomic mutations in SDHx genes. (**B**) Number of patients with mutations in SDHx genes. (**C**) mRNA expression (RSEM values) of mutated vs. non-mutated samples in the four SDH subunits. (**D**) Oncoprint data showing copy number alterations (CNAs) in SDHx genes. (**E**) Number of patients with CNAs in different SDH genes. (**F**) mRNA expression (RSEM values) of altered vs. unaltered samples in the four SDH subunits. (**G**) Oncoprint data displaying mRNA expression of SDHx genes. (**H**) Number of patients with low and high mRNA level in different SDH genes. (**I**) Z score of mRNA expression relative to normal samples for SDH subunits. * = not all samples are profiled.

To complement the findings of differential expression of SDH genes across different breast cancer subtypes from our patient cohort and TCGA data, we have conducted the mutation analysis by using cBioPortal for cancer genomics and investigated the potential driver mutations and genetic alterations in SDH genes in breast cancer. We aimed to elucidate whether driver mutations in SDH genes contribute to the observed expression alterations and whether they may directly influence breast cancer progression. Studies have shown that mutations in metabolic genes can affect tumor biology [53,54,55]. We posited that identifying significant mutations could elucidate the role of these genes as potential drivers of cancer or mere subjects of other regulatory mechanisms. However, the mutation analysis revealed that SDH gene mutations specifically are rare in breast cancer, with only missense or truncated mutations being identified in 0.4% (SDHA) and 0.3% (SDHB) of cases. (Figure 4A,B). We did not detect any mutations in SDHC and SDHD. This result indicates that it is perhaps less likely that observed expression differences in our analyses can be ascribed to missense or truncated mutations in these SDH genes, but rather are more likely a reflection of changes in transcriptional regulation because of copy number alteration or epigenetics. We then analyzed the RNA-Seq by Expectation-Maximization (RSEM) expression values of mutated vs. non-mutated samples for each of these genes. Mutated samples exhibit significantly lower mRNA levels (RSEM gene expression values) than non-mutated samples in the four genes. These results suggest that SDH missense or truncated mutations cause transcriptional repression or instability of affected genes in breast cancer (Figure 4C).

We next examined the copy number changes in the four subunits in the breast cancer samples. While deep deletions primarily impact SDHB and SDHD, amplifications are prevalent in SDHA and SDHC (Figure 4D,E), suggesting a variety of changes within the SDH complex genes. These alterations may impair mitochondrial function and aid in the growth of tumors. Some cancers, such as paragangliomas, are especially linked to loss of SDHB and SDHD [56]. In general, the various mechanisms influencing the course of disease are reflected in the genetic heterogeneity. Side by side, we examined the RSEM mRNA expression values for altered vs. unaltered samples for each of these genes. Altered samples of SDHA and SDHC exhibit significantly higher mRNA levels than the unaltered group, whereas SDHB and SDHD mRNA levels showed no difference between the altered and unaltered groups (Figure 4F). In summary, while altered SDHA and SDHC might reflect transcriptional upregulation as a direct consequence or compensation in cancer, the stability of SDHB and SDHD mRNA levels in altered samples suggests that the underlying alterations are more likely to be epigenetic change at post-transcriptional levels, leading to functional impairment of the SDH complex rather than a change in gene transcription.

Amplification of oncogenes often results in increased mRNA expression, whereas deletion of tumor suppressor genes can lead to decreased mRNA expression [57]. However, it is important to keep in mind that CNAs do not always translate proportionately into altered expression levels due to transcriptional adaptive mechanisms [58]. To clarify more about the alteration, we have examined the mRNA expression data of the TCGA patient cohort. From this analysis we conclude that in most patients, the SDH mRNA (SDHA, SDHB, SDHC and SDHD) level is unchanged. However, SDHC was overexpressed in 43% patients and SDHD was downexpressed in 36% of the total patients (Figure 4G,H). Unlike SDHC and SDHD, the changes affecting SDHA and SDHB are few, with no or only a very moderate frequency of patients showing an altered expression level (Figure 4G,H). The heat map for Z-scores of mRNA expression levels of SDHA, SDHB, SDHC, and SDHD further confirmed the upregulation of SDHC and downregulation of SDHD, while SDHA and SDHB showed neutral expressions (Figure 4I). In summary, most patients commonly exhibit either SDHC overexpression or reduced SDHD expression, whereas the levels of SDHA and SDHB are relatively consistent among patients. These expression changes suggest altered mitochondrial function and metabolic dysregulation. Reduced SDHD levels are often linked to mitochondrial-related diseases and tumor development [17,40,59,60]. Overall, the varied expression patterns highlight distinct roles of SDH subunits in disease processes.

**Figure 5:**
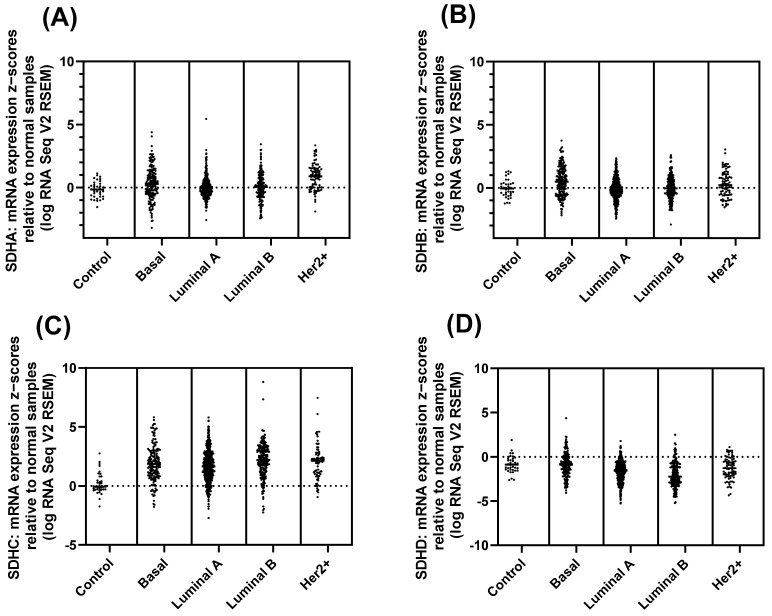
mRNA expression z-score of SDH subunits in breast cancer subtypes. (**A**,**B**) SDHA and SDHB did not alter significantly relative to control. (**C**) SDHC showed overexpression across all subtypes, with a broad distribution of z-scores and many samples displaying positive expression levels. (**D**) In contrast, the majority of samples showed a negative shift in SDHD expression, suggesting that the gene is generally underexpressed compared to normal tissue.

Next, we observed SDH mRNA expression in four different subtypes of breast cancer. The expression of SDH subunits shows a different pattern across breast cancer subtypes. SDHA and SDHB did not significantly alter relative to normal (Figure 5A,B), but SDHC showed higher expression while SDHD showed lower expression in all the subtypes (Figure 5C,D). These findings were further confirmed by mRNA expression RSEM data (Supplementary Figure S3A–D). So, the different ways SDH subunits are expressed in various breast cancer subtypes suggest they might play distinct roles in how these cancers develop, and further study is needed to clarify the mechanisms.

### 2.5. Epigenetic Alteration in SDH Genes

**Figure 6:**
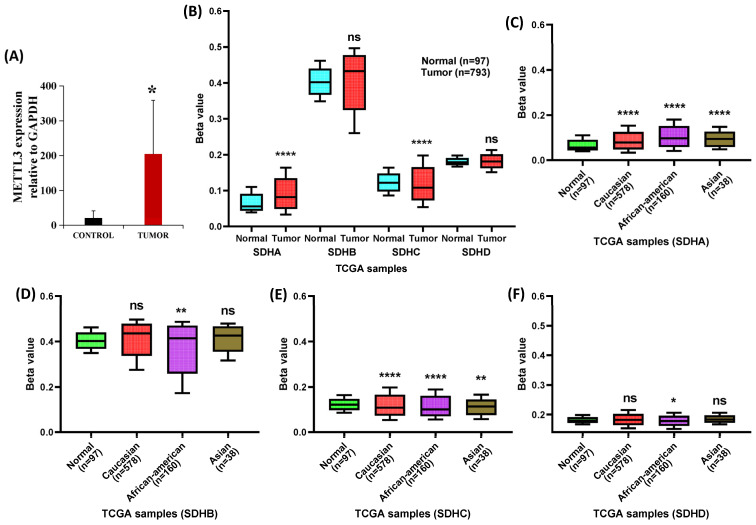
Methylation patterns of SDH subunits. (**A**) Expression of METTL3 in breast cancer patients relative to control. (**B**) Higher beta values are observed for SDHA, SDHB, and SDHD in breast cancer compared to normal tissue, while SDHC shows a lower beta value. (**C**) SDHA methylation is significantly higher across all ethnic groups. (**D**) SDHB methylation is significantly altered only in African Americans. (**E**) SDHC was significantly hypomethylated in all ethnic groups. (**F**) SDHD methylation shows a non-significant increase in Caucasian and Asian groups, but is significantly decreased in African Americans. A *p* value < 0.05 was considered statistically significant. * *p* < 0.05; ** *p* < 0.001; **** *p* < 0.00001; ns, not significant.

Tumor progression is frequently driven by alterations in the m6A “writer” enzyme Methyltransferase-like 3 (METTL3). By upregulating anti-apoptotic Bcl-2 mRNA and suppressing tumor-inhibitory miRNA let-7g, oncogenic METTL3 accelerates the progression of breast cancer [61,62,63,64,65,66]. Thus, METTL3 is a potential diagnostic, prognostic, and therapeutic target in breast cancer due to its association with pathogenesis, poor prognosis, and drug resistance [64]. So, to examine the involvement of m6A-mediated epigenetic alteration, we analyzed the expression of METTL3 in our patient cohort. We observed a significant increase in METTL3 expression in breast cancer tissues compared to adjacent control (Figure 6A). Again, since abnormal DNA methylation of SDH genes is known to be involved in tumor growth, it is important to look at the beta values from genome-wide methylation arrays to compare methylation levels between cancerous and healthy tissues [67,68,69]. To explain the epigenetic alteration in our patient cohort, we compared the beta values of four genes (SDHA, SDHB, SDHC, SDHD) in normal and tumor samples from TCGA dataset. Highest methylation was observed in SDHB among the four subunits (Figure 6B). However, compared to normal samples, higher beta values were observed in SDHA, SDHB, and SDHD, with statistical significance only for SDHA (Figure 6B). In contrast, SDHC showed a significantly lower beta value compared to normal samples (Figure 6B). Subtype-specific methylation analysis showed a differential methylation pattern. SDHA and SDHD methylation was increased in the case of all the breast cancer subtypes (Supplementary Figure S4A,D). On the other hand, SDHC methylation was significantly decreased in all the subtypes (Supplementary Figure S4C). SDHB showed a variations in methylation which increased in Luminal and Her2+ but significantly decreased in TNBC (Supplementary Figure S4B).

We found a significant increases in SDHA gene methylation across ethnic groups, with African American samples exhibiting the highest levels of methylation (Figure 6C). Likewise, SDHB gene methylation is elevated in all populations compared to the normal group, while it is significant only for African Americans (Figure 6D). SDHC gene methylation significantly decreased across populations, with Caucasian and African American groups showing a more pronounced decrease compared to Asian and Normal groups (Figure 6E). The SDHD gene methylation is similar across Caucasian and Asian groups compared to Normal samples. African Americans show a significant decrease in SDHD methylation (Figure 6F). These findings suggest that population-specific epigenetic patterns may exist, potentially influencing disease susceptibility, genetic predispositions, or environmental factors. Understanding these differences could be important for future research into personalized healthcare approaches and disease prevention strategies.

## 3. Discussion

BC is a major global health challenge with significant barriers to effective treatment and prevention due to its complex pathogenesis and wide range of clinical manifestations [70,71]. In order to advance the development of effective therapeutic strategies, it is critical to uncover the complex nature of this devastating disease, as its incidence continues to rise [70]. A crucial connection between energy metabolism, signaling, and illnesses is mitochondrial SDH. The dysregulation of SDH’s subunits, which modifies the metabolism and redox balance of cancer cells, has drawn more attention in the field of cancer research [8,12,15]. Using a patient cohort and publicly available data from TCGA, we examined the expression patterns of all four SDH subunits in breast cancer tissues and their relationship to molecular subtypes of breast cancers and patient survival. The results offer a population-specific perspective on SDH dysregulation and revealed a complex and subtype-specific dysregulation of these genes, which may contribute to breast cancer pathogenesis, reflecting the diversity associated with metabolic reprogramming in cancer [10].

The substantial upregulation of SDHA, SDHB, SDHC, and SDHD in breast cancer tissues versus normal controls is consistent with previous studies that proposed that the dysfunction of SDH can occur frequently in most types of cancers, including breast cancer [24]. Consistent with our findings, it has been reported that disruption of the function of SDH complex results in the reprogramming of metabolism and cancer progression [8,10,31]. But we also found differences in expression among subtypes in Bangladeshi patients, all of which were studied only by us. Although there was variation in TNBC cases, both SDHA and SDHB were elevated in luminal and Her2+ tumors. This variable finding merits additional studies because TNBC is known to be aggressive and has a poorer prognosis [72,73,74,75], and the knowledge of how SDH becomes dysregulated in this subtype could highlight novel therapeutic avenues. SDHC was consistently overexpressed, whereas SDHD expression varied among subtypes and was downregulated in Her2+ cases. This is in line with previous research that suggests SDHD, as a crucial subunit of the SDH complex, might be implicated in tumor suppression [39] and its downregulation may lead to tumorigenesis, especially in Her2+ breast cancers.

Correlations and diverging findings were confirmed by the TCGA database, which expands the differential expression trends in the patient cohort rapidly with higher SDHA, SDHB and SDHC but lower SDHD levels, as detected with patients’ tumors in BC samples. Additionally, TCGA data revealed that SDHC was highly expressed in Luminal, Her2+, and TNBC subtypes, whereas SDHD was downregulated within these three categories. In our series of patients, SDHD was upregulated in Luminal A, Luminal B and TNBC. Furthermore, some discrepancy of SDHA and SDHB expression was observed between our cohort and TCGA dataset. These discrepancies could be due to different experimental protocols, sample size, molecular subtype composition, and epigenetic regulation of clinical samples, particularly considering that the primary patient cohort in the TCGA database is the Western population’s patients, with a larger number of samples contributing to a broader range of distribution of expression values. Furthermore, these diverse expression patterns were further supported by a weak and non-significant correlations between the four SDH subunits, both in our patient cohort and in the TCGA data. This implies that the expression of SDH subcomponents is not strongly co-regulated in breast cancer and can potentially independently drive disease biology. This finding is of significance when considering past studies [6,10,15] that frequently treat genes encoding the SDH complex as a single entity, but our data suggest that there are different regulatory mechanisms or functions for separate subunits in relation to breast cancer.

Impairing mitochondrial metabolism, such as succinate dehydrogenase complex dysfunction, can also generate a build-up of succinate and hypoxia-like signaling, promoting increased tumor aggressive behavior, which then contributes to poor patient outcomes [10]. High expression of SDHA, SDHB and SDHD mRNA is associated with worse overall survival, as we show in our survival analysis, which is also consistent with recent studies [24]. Moreover, low expression of SDHC was associated with adverse survival, a finding consistent with research on basal-like and TNBC breast cancer [39]. This findings would imply a bifaceted relationship in which both overexpression of a few subunits as well as silencing of some could adversely affect patient outcome. The subtype-specific survival analysis showed that the associations were largely consistent with all breast cancer subtypes, but TNBC cases had the worst prognosis of overexpressing all four SDH subunits. This result emphasizes that, in comparison to other subtypes of breast cancer, TNBC is especially vulnerable to SDH dysregulation.

Genetic mutations in these genes may not be the leading cause of expression alterations that we observed here. It is worth noting that our analysis disclosed that mutated samples displayed significantly lower mRNA expression levels than non-mutated ones in all four SDH genes, pointing to an impact of the mutations present on transcriptional inhibition or instability of these genes. Although SDH mutations are rare in breast cancer, it should be mentioned that loss-of-function mutations of SDH subunits have been identified as drivers of other types predisposing to familial paraganglioma syndromes, pheochromocytomas, renal clear-cells, and gastrointestinal stroma tumors, where the genes encoding for the SDH subunits act as tumor suppressor genes [39]. In this regard, SDH mutations are associated with increased stability of HIF-1α and enhanced transcriptional activity of HIF-1, promoting tumorigenesis [31]. The infrequency of SDH mutations and their downregulating impact on mRNA expression in breast cancer suggest a unique regulatory mechanism, distinct from the patterns observed in SDH-deficient tumors in other cancers. In light of the relatively modest role for direct mutation in driving differential SDH expression, our study exposed large deletions in SDHB and SDHD, while amplifications predominated in SDHA and SDHC. These results suggest that copy number alterations in the SDH gene complex could disrupt mitochondrial function and contribute to tumor growth in breast cancer similar to paragangliomas [56].

To comprehend how dysregulation affects breast cancer, it is crucial to examine both CNAs and gene expression [76] because copy number changes may be the cause of SDHC overexpression in certain populations, such as Ghanaian breast tumors [77]. While SDHA and SDHB mRNA levels remained largely unchanged, our analysis of the TCGA cohort revealed downregulation of SDHD and overexpression of SDHC. These differences were all consistent in the breast cancer subtypes studied, whereas SDHA and SDHB did not show significant changes. These results indicate that copy number gains play an essential role in SDHC overexpression, and deletions in SDHD underexpression, leading to dysregulated mitochondrial function and metabolism of breast cancer. We have observed increased expression of METTL3 in breast tissues relative to normal which indicates an METTL3-mediated m6A modification. Although METTL3 was significantly upregulated in our patient cohort, it is important to note that METTL3 represents only one component of a complex epigenetic regulatory network. The observed association between METTL3 expression and SDH subunit methylation is correlative, and no direct causal relationship can be inferred from the present data. Multiple epigenetic regulators are likely to contribute to the observed expression alteration. Further functional and mechanistic studies will be necessary to determine whether METTL3 plays a direct role in regulating SDH subunit methylation.

This study next looked at methylation of SDH genes, and discovered differences in methylation levels between subunits. SDHC showed hypomethylation, but SDHA, SDHB, and SDHD had higher beta values than normal samples where only SDHA showed a significant difference. The hypomethylation of SDHC is consistent with the observation from other studies that tumors with an SDH deficiency may have a tumor suppressive silencing of the SDHC gene, and epigenetic reprogramming could be a hallmark event in these tumors [78,79]. An interesting feature of these results is the discovery of population-specific epigenetic profiles. We found an increase in methylation of the SDHA, SDHB and SDHD genes in Asians compared to normal controls where SDHC significantly decreases the methylation. So, ethnicity-specific epigenome patterns are essential to understand, as they may affect individual risk of diseases due to genetic background or environmental exposure, emphasizing the importance of personalized medicine and disease intervention designed for different ethnic groups.

A major limitation of the present study is the relatively small size of the patient cohort. This limitation may contribute to the differences observed between our cohort and the TCGA database and could reflect sampling variability rather than true biological differences. Consequently, these findings should be interpreted with caution. Larger, independent cohorts will be required to validate these observations and to determine their broader applicability. Furthermore, we focused our analysis on SDH subunits and METTL3 to specifically examine SDH-related dysregulation and its possible epigenetic regulation in breast cancer, while acknowledging that broader transcriptomic approaches could uncover additional metabolic pathways and should be explored in future studies. Above all, the observed upregulation of SDHD in the Bangladeshi cohort compared to its downregulation in TCGA samples may be influenced by several confounding factors beyond biological variation. Differences in sample preservation methods, RNA extraction and normalization methods variations, RNA quality, cohort ancestry composition, and underlying clinical heterogeneity, including age distribution, disease stage, and treatment history, could contribute to the divergent expression patterns.

## 4. Materials and Methods

### 4.1. Study Design, Ethical Consideration, and Participant Information

The current study was conducted on twenty-five breast cancer cases that were diagnosed and admitted to the Department of Oncology at Chattogram Medical College Hospital, Bangladesh. All the participants signed the informed consent before sampling. The study was approved by the Institutional Review Board of CMCH. Patients were selected based on the inclusion criteria. The criteria were confirmed diagnosis of breast cancer by IHC, record of mastectomy or breast conserving surgery (BCS) and patients with no other comorbidity. A face-to-face interview was conducted using a structured questionnaire to collect detailed data on age at diagnosis, socioeconomic status, family history, medical history, lifestyle, and biophysical parameters.

### 4.2. Tissue Sample Collection and Preservation

The malignant and adjacent histologically normal breast tissues were surgically resected by a general surgery surgeon in CMCH as part of the patient’s therapy plan. The excised tissues were immediately immersed in RNA later stabilization solution and stored at −20 °C as a temporary measure until they were finally stored at −80 °C. The present tissue samples were used for analysis of succinate dehydrogenase expression [80].

### 4.3. Histopathology and Immunohistochemistry of Breast Tumor Samples

Histopathology and immunohistochemistry were used to confirm malignancy and to identify the tumor grading, staging, and typing. The adjacent non-cancerous tissues were also examined via histopathology to confirm the cellular and tissue architecture of healthy cells. Evaluation was done by a certified pathologist before inclusion in the study. The existence and specific localization of BC biomarkers such as estrogen receptor (ER), progesterone receptor (PR), and human epidermal growth factor receptor 2 (Her2) were verified by IHC. Specifically, primary antibodies included EP1 for ER, PgR636 for PR, and an anti-human c-erbB2 oncoprotein antibody for Her2/neu [81].

### 4.4. Extraction and Quantification of RNA

Total RNA was extracted from tissue using a chloroform–isopropanol method. Initially, the tissue samples were minced and washed in PBS. Then TRIzol was added, and the mixture was homogenized. Immediately, the lysate was placed in a microtube with 1.5 mL of chloroform, vortexed, and centrifuged for 20 min at 15,000 rpm to create a non-polar organic layer and a polar aqueous layer. The RNA comprising the aqueous layers was taken and then precipitated using an isopropanol-saturated solution. Following the precipitation, the precipitate was washed with 75% ethanol, air-dried, and then dissolved in RNase-free water. The concentration and purity of RNA were measured using a NanoDrop ND-2000 spectrophotometer (Thermo Fisher Scientific, Waltham, MA, USA) at 260 nm and 280 nm. The A260/A280 ratios, ranging from 1.6 to 1.8, indicated good RNA purity and acceptable RNA purity for gene expression analysis [82].

### 4.5. Agarose Gel Electrophoresis

Agarose gel electrophoresis was also performed to verify that the RNA size was correct. The gel was made using (1× Tris-Borate-EDTA) buffer and staining was done using ethidium bromide (EtBr). RNA samples were mixed with 6× loading dye and then electrophoresed in the electrophoresis chamber. Hence, the band observed indicates that it is of acceptable quality for further use in qRT-PCR downstream processes.

### 4.6. cDNA Synthesis from Total RNA

For the synthesis of complementary DNA (cDNA), total RNA was normalized to 500 ng/µL and was reverse transcribed using the Promega corporation’s GoScript™ (Promega Corporation, Madison, WI, USA) reverse transcription system. The synthesis involved priming with Oligo (dT), followed by the addition of the GoScript™ master mix and reverse transcriptase. The optimized thermal cycling conditions were used in this protocol. Finally, the concentration of the cDNA was quantified using a NanoDrop spectrophotometer [82].

### 4.7. Oligo Designing for Target-Specific Real-Time PCR

Target-specific oligo design was carried out through NCBI database and Integrated DNA Technologies. Our designed primer sequences are mentioned in Table 1. The primer was selected by using near about 18–30 nucleotides in length with 40 to 60% of GC content from the gene selected. The melting temperature is at almost 50–60 °C and the product length is selected from 100 to 300. We avoided complementarity between forward and reverse sequence during the design of a forward and a reverse primer for both the targeted genes and the reference gene.

### 4.8. Quantitative RT-PCR Analysis

The relative expression of SDHA, SDHB, SDHC, and SDHD was determined using quantitative reverse transcription PCR. The GoTaq qPCR master mix was used to assay the reaction in a real-time PCR system. The reaction consisted of cDNA, gene-specific primers and master mix, and was run in triplicate for three independent experiments. The reaction mixture was incubated in Applied Biosystem instruments at 95 °C for 2 min, followed by 40 cycles at 95 °C for 15 s, 58–60 °C for 30 s, and 72 °C for 30 s [83]. A melt curve analysis was performed after the 40 cycles to confirm the specificity of the product. The GAPDH was used as a control and the relative expression levels of SDHA, SDHB, SDHC and SDHD were determined using the 2^−ΔΔCt^ method to compare the expression of tumor and adjacent non-tumor tissues [84].

### 4.9. Analysis of TCGA Dataset

The Cancer Genome Atlas (TCGA) was utilized to validate the mRNA expression levels of SDHA, SDHB, SDHC and SDHD. The TCGA delivered a comprehensive molecular portrayal of more than 20,000 primary tumors from 33 cancers, involving genomic, transcriptomic, proteomic, epigenomic, and clinical data. Moreover, the data mining applications were utilized in the study, including the UALCAN and cBioPortal, which combine the gene expression, proteomic, and survival prognosis analysis [85].

### 4.10. Survival Analysis

The survival analysis was performed as described previously [86] to assess overall survival among breast cancer patients. The Kaplan–Meier plotter is an online bioinformatics analysis system used to determine the prognostic value of gene expression. We have done a 240-month follow-up analysis to check the high and low levels of SDHA, SDHB, SDHC, and SDHD in breast cancer patients.

### 4.11. Statistical Analysis

Statistical analysis was done using Microsoft Excel and GraphPad Prism 8. Microsoft Excel was used to determine the mean ± SD. A two-tailed unpaired *t*-test was used to compare the tumor group and the control group. A value of *p* < 0.05 was considered statistically significant.

### 4.12. Materials

All reagents and consumables used in this study were purchased from reputed manufacturers to ensure the accuracy and repeatability of the analysis. The EDTA tube used for blood collection was obtained from Qiagen (Germantown, MD, USA). RNA extraction was done by using Trizol TM Reagent from Thermo Fisher Scientific, Waltham, MA, USA. Molecular-grade agarose was procured from Sigma-Aldrich (St. Louis, MO, USA). Other reagents, such as DNase/RNase-free water, 5× RT reaction buffer, a reverse transcriptase enzyme, 100 mM dNTPs, RNase inhibitor (20 U/µL), qPCR Mastermix, Taq polymerase enzyme kit, and the Super Two-Step RT-PCR system SYBR Green, were all purchased from Promega Madison, WI, USA. Chloroform and ethanol were acquired from Merck (Darmstadt, Germany), Isopropanol was purchased from BDH Chemicals Ltd. (Radnor, PA, USA), and EtBr was collected from Bioshop (Burlington, ON, Canada).

## 5. Conclusions

Our findings show a distinct profile of SDH dysregulation in Bangladeshi breast cancer patients, with all four SDH subunits continuously upregulated, including the noticeably elevated SDHD that deviates from TCGA patterns. Subtype-specific differences further emphasize the metabolic diversity of breast cancer. Furthermore, elevated SDHD expression has been associated with poorer patient outcomes, suggesting a contributory role in cancer progression. We revealed that both genetic and epigenetic modifications, such as copy number variations and DNA methylation, are responsible for the disruption of SDH expression. The significant rise in METTL3 expression suggests a m6A-dependent epigenetic mechanism that contributes to SDHD activation. These findings highlight the need for population-specific studies of tumor metabolism and suggest SDHD as a previously unidentified metabolic and epigenetic marker in breast cancer. Finally, our findings emphasize how important it is to consider both genetic and epigenetic mechanisms to understand the biology of breast cancer and create more customized treatment plans.

## Figures and Tables

**Table 1:** Oligo primer sequence of the genes.

Gene Symbol	Primer	Sequence (5′→3′)
*SDHA*	F	CCAGGAATGGTCTGGAACAC
	R	GAGAAGGCCCACCTTGTAGT
*SDHB*	F	GCAGTATCTGCAGTCCATAG
	R	CGATAGGCCTGCATAAGAAC
*SDHC*	F	CTGTTGCTGAGACACGTTG
	R	CAGAGGACGGTTTGAACCTA
*SDHD*	F	CCTGATGCTGATCTGACAATGG
	R	GTACAGAAAGGAGGGCAGTAG
*GAPDH*	F	CAGCCTCAAGATCATCAGCA
	R	TGTGGTCATGAGTCCTTCCA
*METTL3*	F	CCAGCACAGCTTCAGCAGTTCC
	R	GCGTGGAGATGGCAAGACAGATG

F, forward primer; R, reverse primer.

## Data Availability

The original contributions presented in this study are included in the article/Supplementary Material. Further inquiries can be directed to the corresponding author.

## Supplementary Materials

The following supporting information can be downloaded at: https://www.mdpi.com/article/10.3390/ijms27041722/s1.

## Abbreviations

The following abbreviations are used in this manuscript:

BC	Breast cancer
BCS	Breast conserving surgery
BMRC	Bangladesh Medical Research Council
cDNA	Complementary DNA
CNAs	Copy number alterations
ER	Estrogen receptor
EtBr	Ethidium bromide
Her2+	Human epidermal growth factor Receptor 2 positive
IHC	Immunohistochemistry
METTL3	Methyltransferase-like 3
PR	Progesterone receptor
RSEM	RNA-Seq by Expectation-Maximization
SDH	Succinate dehydrogenase
TCGA	The Cancer Genome Atlas
TNBC	Triple-Negative Breast Cancer
