# Supplementary material for: Altered Expression of Mitochondrial Succinate Dehydrogenase Subunit D Influences Breast Cancer Progression
Source: Int J Mol Sci. 2026 Feb 11;27(4):1722. doi: 10.3390/ijms27041722 (PMC12940868; doi:10.3390/ijms27041722)
Supplement: Supplementary file 1 [file ijms-27-01722-s001.zip › ijms-4069799-supplementary.pdf]

Supplementary Material

1. Supplementary Figure S1

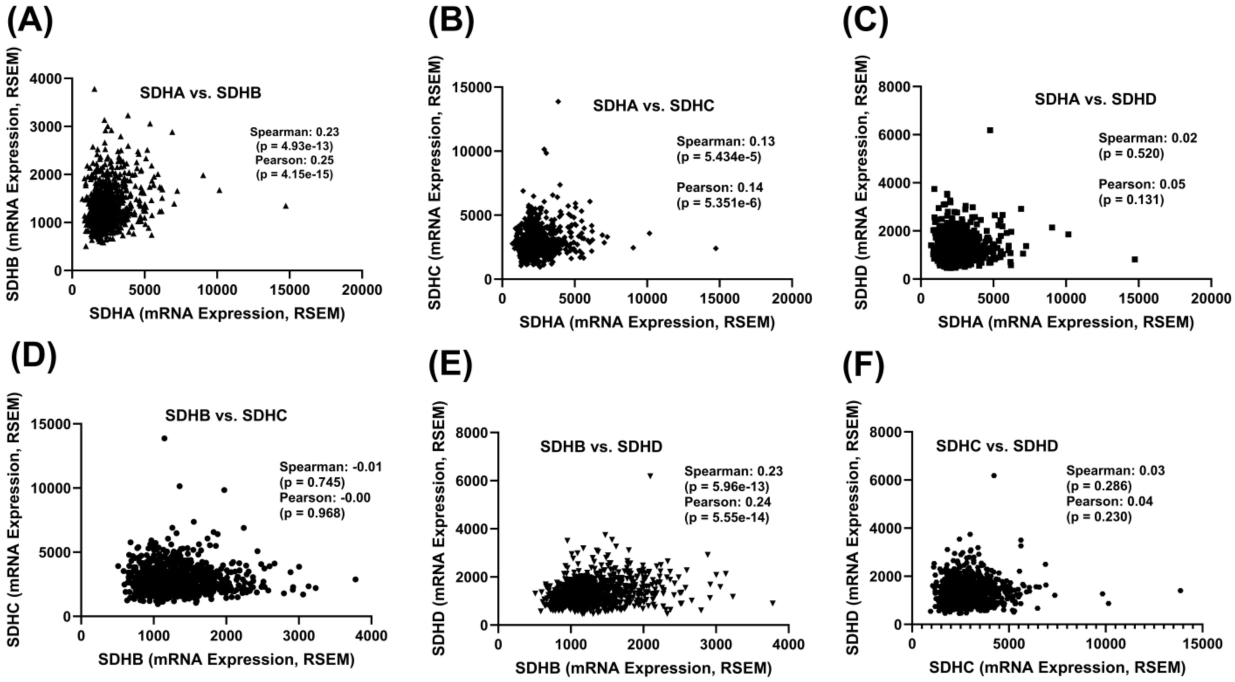

Supplementary Figure S1. Correlations of mRNA expression (RSEM) among SDH subunits. (A) SDHA vs. SDHB shows a weak positive correlation. **(B)** SDHA vs. SDHC also demonstrates a weak correlation. **(C)** SDHA vs. SDHD exhibits minimal correlation. **(D)** SDHB vs. SDHC shows no significant correlation. **(E)** SDHB vs. SDHD displays a weak positive association. **(F)** SDHC vs. SDHD exhibit minimal correlation. Each scatter plot shows individual sample values, with SDH subunit expression plotted on the x- and y-axes as indicated.

2. Supplementary Figure S2

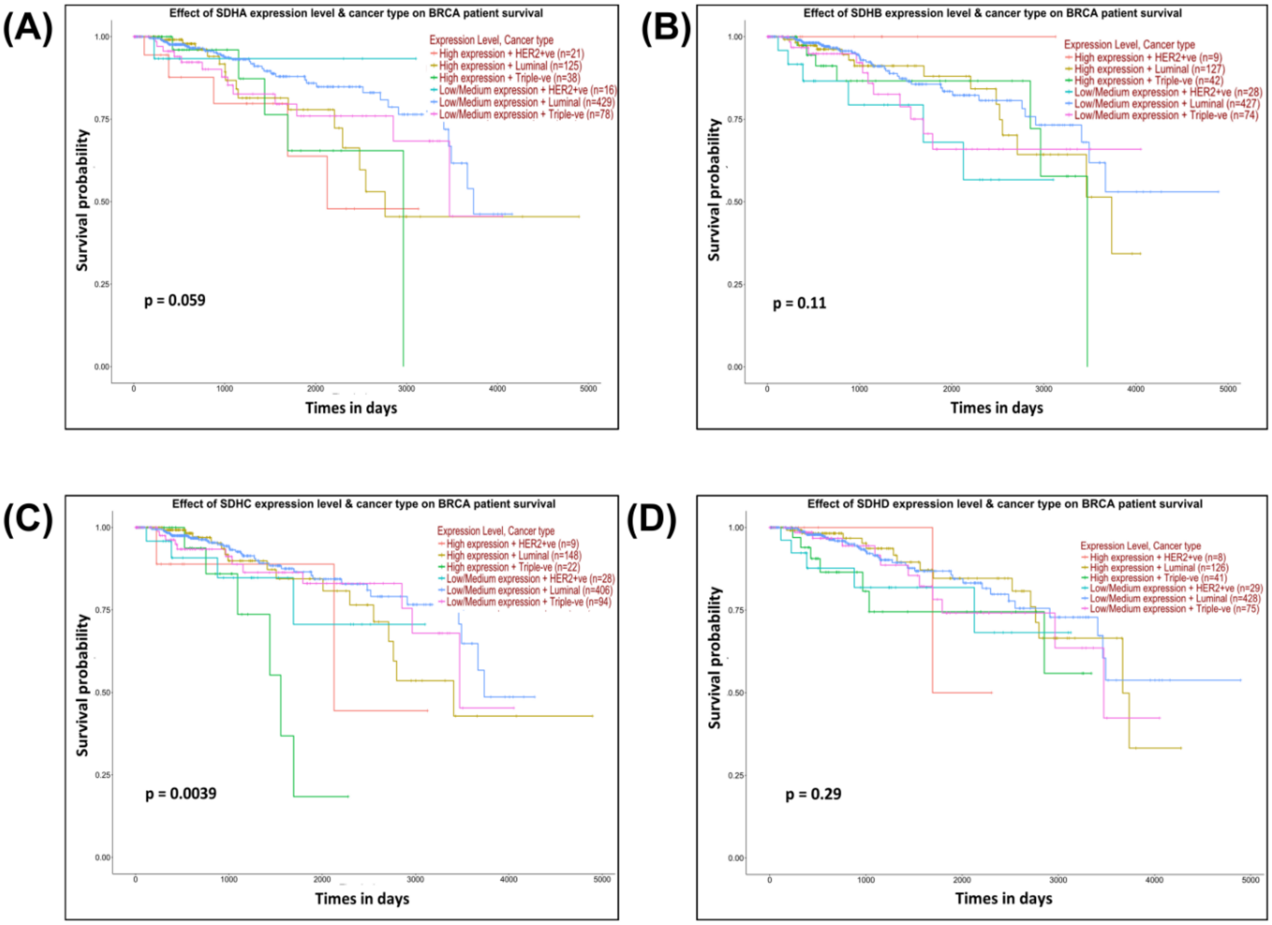

Supplementary Figure S2. Subtype specific survival analysis. The Kaplan-Meier plotter was used to assess overall survival probability in patients. **(A)** Higher expression of SDHA was associated with shorter overall survival in all subtypes. **(B)** Similar observation for SDHB, except in the HER2+ subtype, where high SDHB expression showed no significant change in overall survival. **(C)** For SDHC, the lower expression group exhibited longer overall survival. **(D)** Higher expression of SDHD was linked to lower overall survival in all subtypes.

3. Supplementary Figure S3

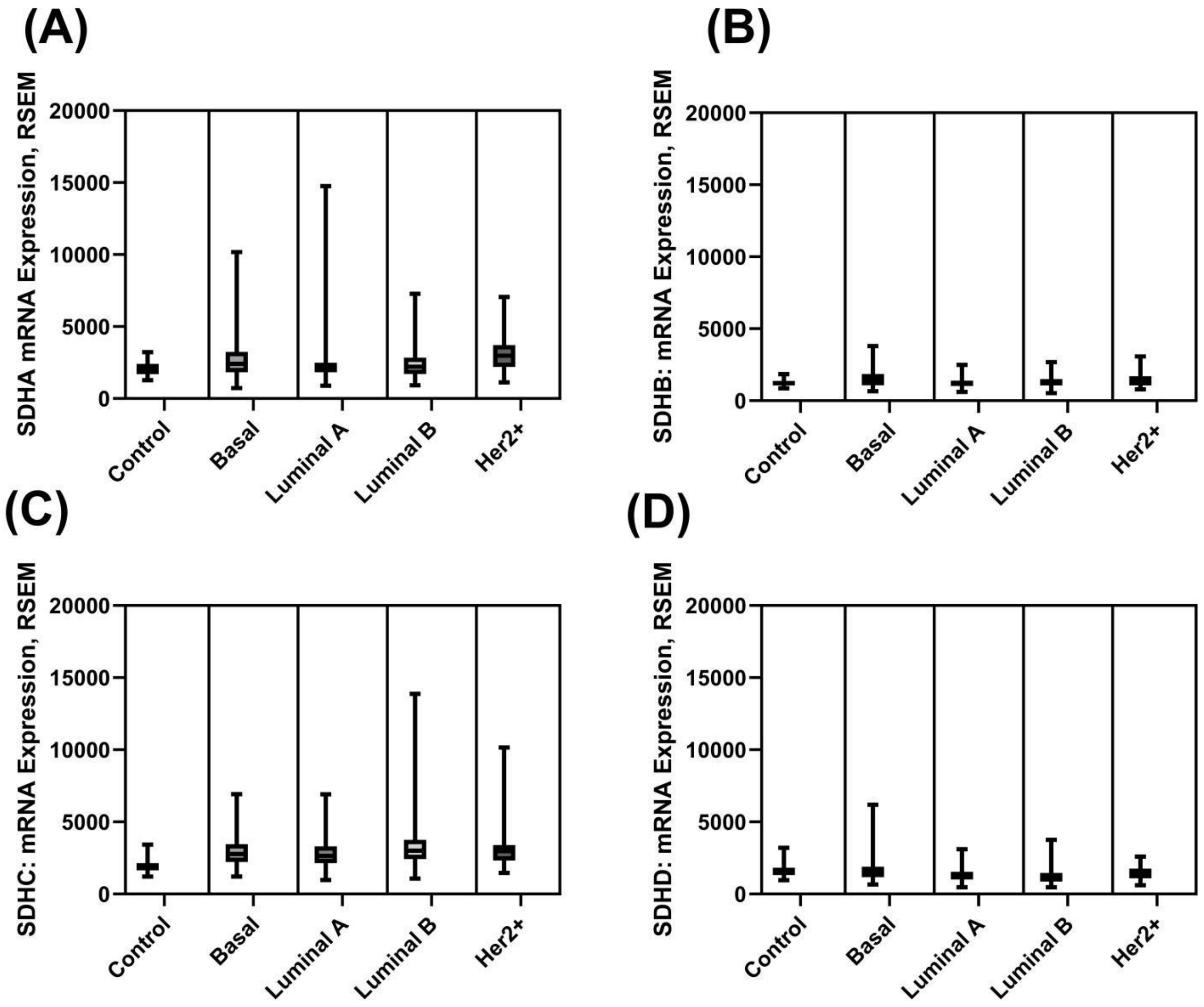

Supplementary Figure S3. Differential expression of SDH subunits in breast cancer subtypes. RSEM-normalized mRNA expression levels of **(A)** SDHA, **(B)** SDHB, **(C)** SDHC, and **(D)** SDHD in Basal, Luminal A, Luminal B, and Her2+ breast cancer subtypes, compared to normal controls. The plots display expression distributions within each subtype, with whiskers indicating variability and range.

4. Supplementary Figure S4

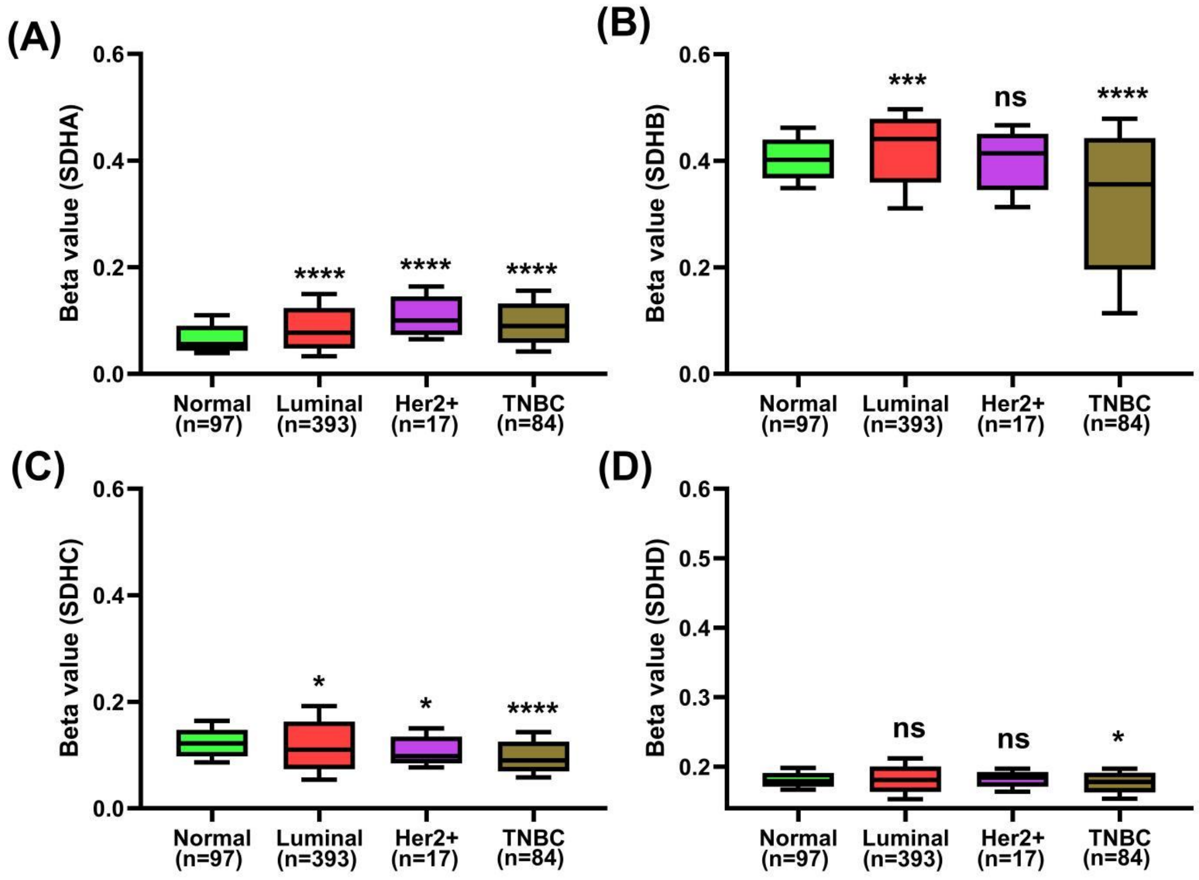

Supplementary Figure S4. Subtype specific methylation patterns of SDH subunits. Methylation patterns of **(A)** SDHA, **(B)** SDHB, **(C)** SDHC, and **(D)** SDHD in four breast cancer subtypes from TCGA datasets. The total number of patients for each group is as follows: normal (97), Luminal A (393), Her2+ (17), and TNBC (84). Beta values were used to calculate SDH gene methylation. A P value < 0.05 was considered statistically significant.
